# Identification and Characterization of Mycobacterium tuberculosis Beijing Genotype Strain SBH163, Isolated in Sabah, Malaysia

**DOI:** 10.1128/MRA.01322-19

**Published:** 2020-01-09

**Authors:** Jaeyres Jani, Siti Fatimah Abu Bakar, Zainal Arifin Mustapha, Chin Kai Ling, Roddy Teo, Kamruddin Ahmed

**Affiliations:** a Borneo Medical and Health Research Centre, Faculty of Medicine and Health Sciences, Universiti Malaysia Sabah, Kota Kinabalu, Sabah, Malaysia; b Department of Information Systems, School of Computing, Faculty of Engineering, Universiti Teknologi Malaysia, Skudai, Johor, Malaysia; c Department of Medical Education, Faculty of Medicine and Health Sciences, Universiti Malaysia Sabah, Kota Kinabalu, Sabah, Malaysia; d Department of Pathobiology and Medical Diagnostics, Faculty of Medicine and Health Sciences, Universiti Malaysia Sabah, Kota Kinabalu, Sabah, Malaysia; e Tuberculosis and Leprosy Control Unit, Sabah State Health Department, Kota Kinabalu, Sabah, Malaysia; Georgia Institute of Technology

## Abstract

This is a report on the whole-genome sequence of Mycobacterium tuberculosis strain SBH163, which was isolated from a patient in the Malaysian Borneo state of Sabah. This report provides insight into the molecular characteristics of an M. tuberculosis Beijing genotype strain related to strains from Russia and South Africa.

## ANNOUNCEMENT

The situation regarding tuberculosis in Sabah state in Malaysian Borneo, where 20% to 30% of the total number of cases in Malaysia occur, is alarming (1). Although the spread of strains from immigrants is perceived to be a factor in the large number of tuberculosis cases (2), the exact reasons for this are unknown. To explore this further, whole-genome sequencing (WGS) of Mycobacterium tuberculosis strains isolated from patients was performed. This paper reports an M. tuberculosis strain of the Beijing genotype that was isolated in Sabah. The Beijing genotype is widespread and is becoming prevalent worldwide. Many studies have indicated Beijing genotype strains as a concern, since strains of this genotype may have a predilection to develop drug resistance (3).

M. tuberculosis strain SBH163 was isolated from the sputum of a 43-year-old male patient from Kota Belud, Sabah, Malaysia. The patient was diagnosed as positive for M. tuberculosis through GeneXpert MDR/RIF testing (Cepheid). The strain was cultured in 7H9 Middlebrook medium using the Bactec MGIT 320 system (Becton, Dickinson). Genomic DNA was subsequently extracted using the MasterPure complete DNA and RNA purification kit (Epicentre). The quality of the extracted DNA was determined with a NanoDrop 2000c spectrophotometer, and the concentration was ascertained using a Qubit 2.0 fluorometer (Thermo Fisher Scientific).

WGS was performed with the Illumina HiSeq 4000 platform, and the sequencing generated 11,040,284 paired-end 150-bp reads. The quality of the sequence reads was checked using FastQC (https://www.bioinformatics.babraham.ac.uk/projects/fastqc). All of the raw reads were preprocessed using BBMap v.38.43 tools. The raw sequence data were checked for quality (Phred score of Q30) prior to trimming to ensure high-quality data for the assembly process. The sequence coverage was 253×. *De novo* assembly was completed using SPAdes v.3.11.1, with default parameters (3), and generated 102 contigs, with a genome size of 4,355,733 bp and an *N*_50_ value of 130,711 bp. The GC content of the genome was 65.59%. The assembled genome from the combined data set was annotated with the NCBI Prokaryotic Genome Annotation Pipeline (4), which yielded 4,299 predicted genes.

**FIG 1:**
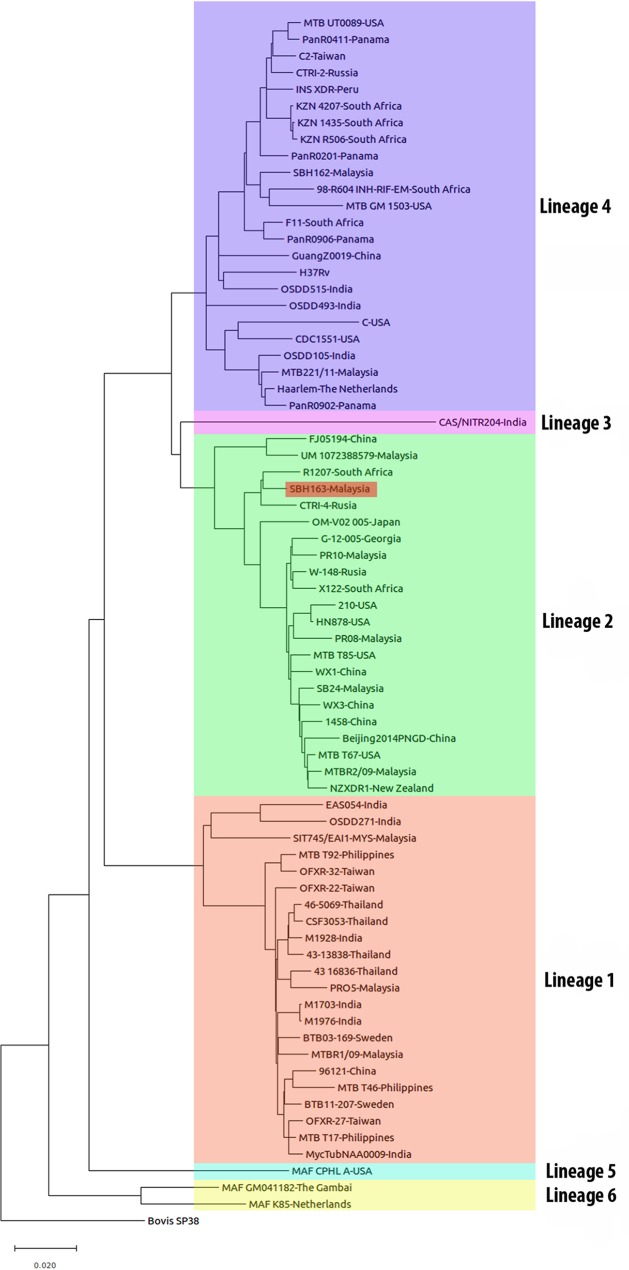
Comparative phylogenetic analysis of strain SBH163. This strain belongs to lineage 2 and is clustered with Beijing genotype strains from South Africa and Russia but not with Beijing genotype strains from Sabah (strain SB24) and other parts of Malaysia. The phylogenetic tree was constructed using SNP data extracted from the genome sequences. The phylogenetic tree was inferred using the maximum likelihood method and a GTR model. Mycobacterium bovis strain SP38 was used as an outgroup.

A total of 72 genomes of M. tuberculosis strains (5–8) were extracted from GenBank, and core genome single-nucleotide polymorphisms (SNPs) were identified using kSNP3 tools (9). The entire SNP matrix was used for phylogenetic analysis, using the maximum likelihood method available in MEGA v.6.0 (10). The analysis used a general time-reversible (GTR) model. The tree showed that our strain belonged to the Beijing genotype of the East Asian lineage (lineage 2) (11) and clustered with strain CTRI-4 from Russia (12) and strain R1207 from South Africa (13) (Fig. 1). Until now, WGS results for only two M. tuberculosis strains from Sabah have been reported; these are strain SBH162, which belongs to the Latin American-Mediterranean (LAM) family of lineage 4 and was isolated from the sputum of a patient with pulmonary tuberculosis (8), and strain SB24, which belongs to the Beijing genotype of lineage 2 and was isolated from the cerebrospinal fluid of a patient with tuberculous meningitis (14). However, the two Beijing genotype strains from Sabah were in different clusters on the phylogenetic tree, which indicated their origins from different ancestors. WGS of more strains would be necessary to elucidate the true picture of circulating Beijing genotype strains in Sabah.

### Data availability.

Raw reads were deposited in the NCBI SRA under accession no. SRR10209206. Data for this whole-genome shotgun project were deposited in DDBJ/ENA/GenBank under accession no. WFLD00000000.
